# Comprehensive Morpho-Functional and Dental Rehabilitation of a Complete Unilateral Cleft Lip and Palate Patient

**DOI:** 10.7759/cureus.21043

**Published:** 2022-01-09

**Authors:** Rizwana Mallick, Sweta K Pisulkar, Srinivas G Reddy, Vanshika Jain

**Affiliations:** 1 Department of Prosthodontics and Crown & Bridge, Jamia Millia Islamia, Delhi, IND; 2 Department of Prosthodontics and Crown & Bridge, Sharad Pawar Dental College, Wardha, IND; 3 Department of Oral and Maxillofacial Surgery, GSR Institute of Craniomaxillofacial and Facial Plastic Surgery, Hyderabad, IND; 4 Department of Oral Medicine and Radiology, Government Dental College and Research Institute Bangalore, Bengaluru, IND

**Keywords:** patient care team, orofacial cleft, mouth rehabilitation, immediate dental implant loading, alveolar bone grafting

## Abstract

Congenital clefts cause compromised function and esthetics, inadvertently affecting a patient’s social and mental health. These defects can be successfully managed by a multidisciplinary team that can provide holistic care from birth till adulthood and beyond. A 17-year-old girl with left side congenital cleft, who had undergone cleft surgeries at our center, reported with a chief complaint of a missing front tooth in the upper region. Clinical and radiographic investigations showed a need for tertiary grafting, which was done using an autologous iliac graft. After six months, a dental implant was placed and immediately loaded after implant stability quotient assessment. Treatment of cleft patients is arduous and technique sensitive and should be done following pre-defined protocols. Each case should be handled by a multidisciplinary team giving attention to each aspect of the treatment requirement. It is an added advantage if the treatment is holistically catered at a single center as it provides patient comfort and avoids patient dependence on past records.

## Introduction

Congenital orofacial clefts affect one in 500-2,500 newborns globally [1,2], having individual or combination, unilateral or bilateral, and incomplete or complete involvement of lip, alveolus, and hard and soft palate. These defects cause problems like difficulty in feeding and swallowing, nasal and oral regurgitation, delayed speech development, improper phonetics and hearing difficulties, facial disfigurement, and dental problems like malaligned or malformed teeth, with either hypo or hyperdontia [2,3]. To minimize these challenges, patients undergo technique-sensitive and time-consuming surgeries from an age of three to six months in adjunct with non-surgical treatments like psychological consultations and speech therapy sessions, which extend till adulthood [3]. Thus, it is important that a carefully curated protocol addressing all treatment needs is followed and preferentially provided at a single center, thus increasing the treatment success by manifolds.

This case report was previously presented as faculty scientific paper presentation at the 49th Indian Prosthodontic Society National Virtual Conference 2021 on December 1, 2021, and won the best paper of the session award.

## Case presentation

A 17-year-old female visited our center with a chief complaint of a disfigured nose and missing tooth in the upper front region. The patient desired a fixed replacement as the missing tooth caused her psychological distress and esthetic insecurity.

**Figure 1:**
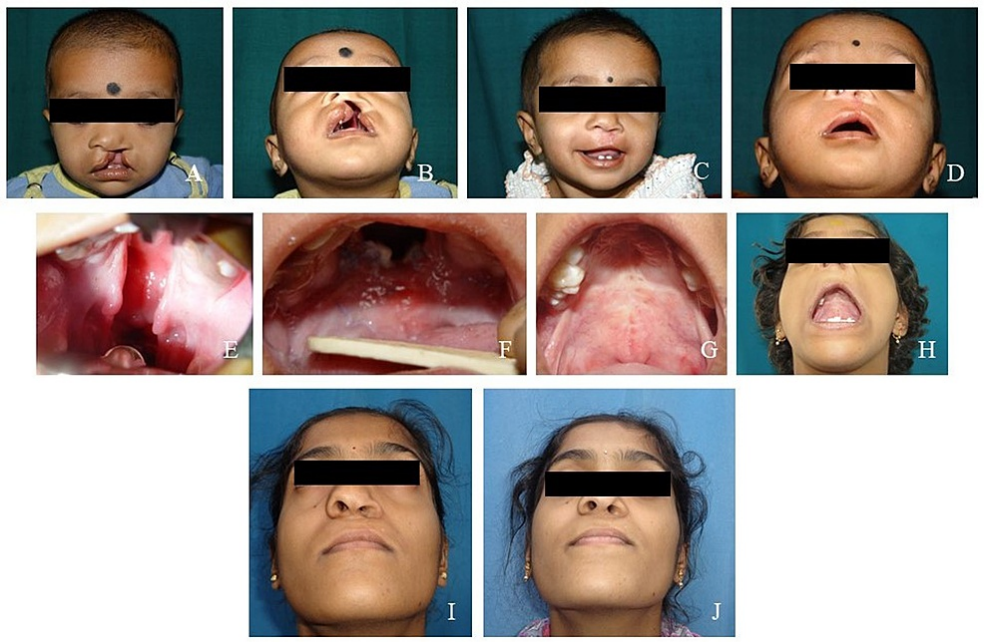
A: Patient at six months with unoperated clefts. B: After repair of the cleft lip at six months. C: Patient's esthetics after repair of cleft lip (front view). D: Patient's esthetics after repair of cleft lip (worm’s eye view). E: Unoperated cleft of the palate. F: After repair of the cleft of the soft palate at one-and-a-half years. G: After repair of the cleft of the hard palate at two-and-a-half years (intra-oral). H: After repair of the cleft of the hard palate at two-and-a-half years (worm’s eye view). I: Patient at 17 years before tonsillectomy. J: Patient at 17 years after tonsillectomy.

Born in June 2002 with a complete unilateral left-sided cleft involving the lip, alveolus, and hard and soft palate (Figures 1A, 1B), the girl was surgically and non-surgically treated at our center. In March 2003 at nine months of age, a morpho-functional cleft lip repair was the first cleft-related surgical procedure performed (Figures 1C, 1D). At ages of one-and-a-half and two-and-a-half years, respectively, second and third surgical procedures involving repair of soft and hard palate clefts were done using Delaire two-stage functional palatoplasty (Figures 1E-1H). Nutritionist consultation since beginning with speech therapy sessions at two-and-a-half years was started to avoid malnutrition and ensure proper development of phonetics.

At six-and-a-half years of age in 2009, the patient revisited the center for secondary alveolar bone grafting (SABG), which later allowed eruption of permanent maxillary canine in the cleft region. In 10 years period till 17 years of age, orthodontic treatment without cleft-related surgical interventions was done by which best possible occlusion was achieved; however, the malformed tooth did not allow adequate space closure between the left central incisor and canine. At 17 years of age, rhinoplasty was done in May 2019 to address the chief complaint of the disfigured nose, which also reduced episodes of nasal congestion faced by the patient (Figures 1I, 1J).

**Figure 2:**
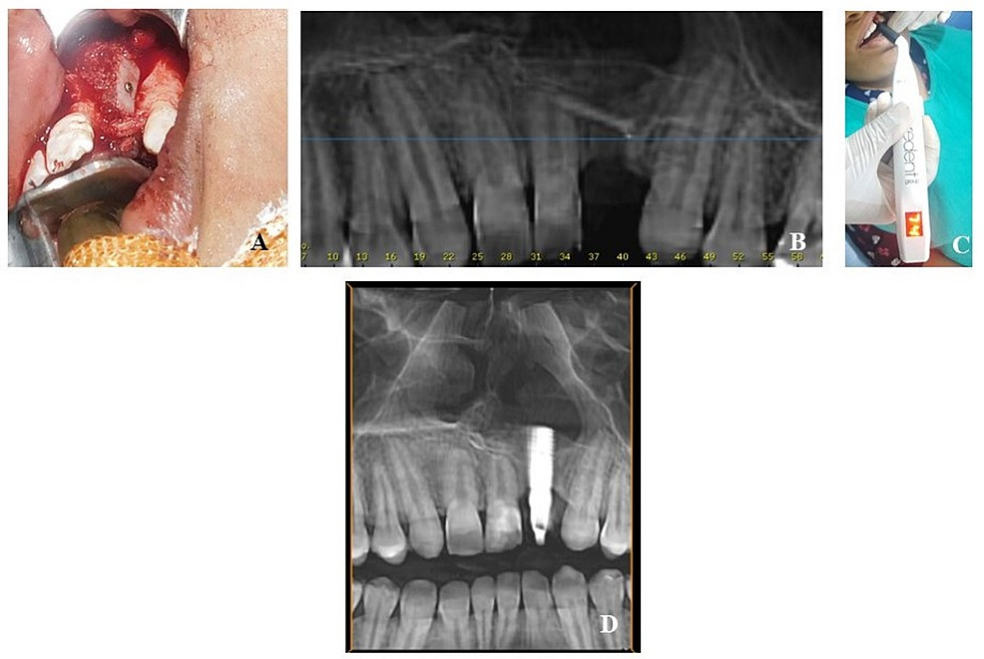
A: Iliac graft stabilized in defect using titanium screw during tertiary grafting. B: Cone-beam computed tomography scan recorded six months after grafting. C: Primary ISQ recording. D: CBCT recorded after implant placement. CBCT, cone-beam computed tomography.

Clinical and radiographic assessments done after six months confirmed the presence of malformed permanent left lateral incisor with external root resorption, making it non-salvageable. It also showed inadequate bone for dental implant thus requiring tertiary alveolar grafting. At 18.5 years of age in December 2020, tertiary grafting was done using a combination of autogenous corticocancellous anterior iliac bone graft and platelet-rich fibrin. Cortical perforation of the recipient site was performed followed by graft beveling and adaptation, which was stabilized using titanium screws (Figure 2A). The defect was slightly overcompensated accounting for inadvertent graft resorption. Post-operative follow-ups showed no complications with satisfactory surgical sites healing. Bone regeneration was confirmed using a cone-beam computed tomography (CBCT) scan recorded six months after grafting, which presented an Enemark score of 2 (Figure 2B).

A 4.0 x 1 2 mm titanium dental implant (TitanGrade 4, blasted etched implant surface, Bredent GmbH Co, Senden, Germany) was placed in grafted site at an insertion torque of 30 Ncm. Primary implant stability quotient (ISQ) was assessed using Penguin RFA device (Integration Diagnostics Sweden AB, Gothenburg, Sweden), which showed a value of 74 (Figure 2C), thus enabling immediate loading, which was done using cement-retained polymethyl methacrylate temporary prosthesis. A CBCT was also recorded at this stage to act as a base level recording (Figure 2D).

**Figure 3:**
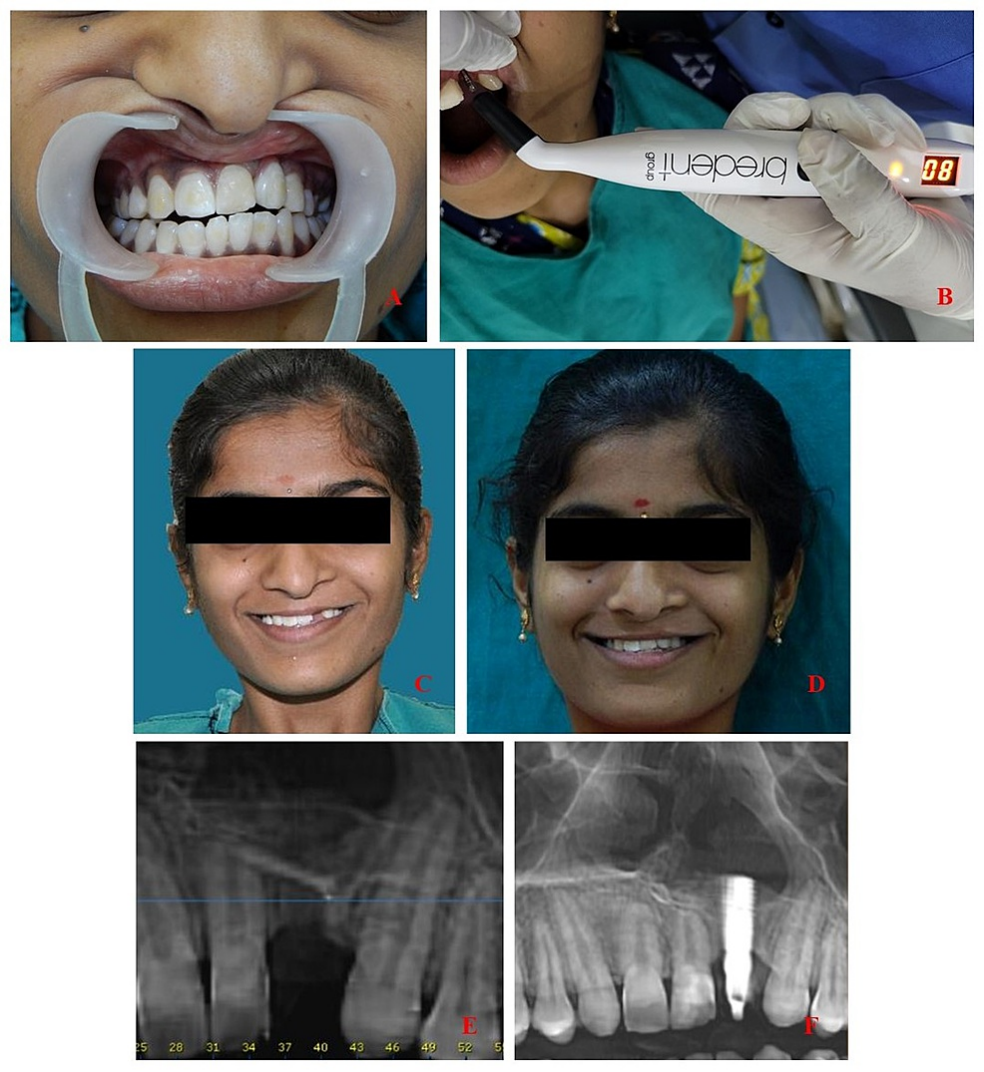
A: Definitive prosthesis in place. B: ISQ assessment prior to definitive crown cementation. C and D: Improvement in patient’s esthetics following dental implant-based rehabilitation. E and F: Pre- and post-operative CBCT images. ISQ, implant stability quotient; CBCT, cone-beam computed tomography.

Three months after immediate loading, the temporary prosthesis was replaced with a screw-retained definitive porcelain-fused-to-metal crown (Figure 3A). Before its placement, ISQ was re-assessed, which was an enhanced value of 80 (Figure 3B). At this point, the implant did not show mobility with the presence of healthy gingival tissue and universal probing depths of 2-3 mm. Initial osseointegration of the dental implant was evident in the recorded CBCT, which showed no radiolucency at the implant-bone surface. Enhanced osseointegration was evident in the increased ISQ value recorded three months later at the time of definitive prosthesis placement.

The patient was successfully rehabilitated using a dental implant, which was immediately loaded thus decreasing treatment time. This significantly improved patient’s esthetics and helped address her chief complaints (Figures 3C-3F). The surgical procedures done during infancy till the age being a young adult along with orthodontic intervention, speech therapy sessions, and nutritionist support helped overcome the problems of feeding, nasal regurgitation, jaw development, and improved facial esthetics, and ensured the proper development of phonetics and healthy growth. The patient was satisfied with the last performed surgical procedure of rhinoplasty and rehabilitation of the missing tooth, which improved her esthetics and increased her confidence. All mentioned surgical and non-surgical procedures including dental rehabilitation were performed at a single cleft care center.

## Discussion

India sees numerous congenital cleft cases with a comparatively smaller number of comprehensive care centers. Each case is a unique presentation and requires sequential treatment for prediction of outcomes and potential complications, providing scope for modifications. A lack of a universal treatment approach was found by de Ladeira and Alonso who highlighted the absence of evidence-based surgical cleft treatment [4]. Repairing soft palate before hard palate prevented growth inhibition subsequently negating the need for additional surgical procedures like LeFort osteotomy [5]. Iliac bone graft was preferred over symphyseal graft because of the availability of larger graft volume and its corticocancellous nature. It also allowed simultaneous operations, decreasing intra-operative time, and avoided the potential risk of tooth devitalization and mental nerve injury associated with mandibular graft [6]. Recipient site perforation helped induce angiogenesis and early graft integration with the host site. The literature presents scarce data concerning the same; however, positive results, in this case, encourage further exploration of surgical techniques [7,8]. Undertaking tertiary grafting not only helped achieve the required bone dimension for implant placement but its subsequent loading using a dental implant will ensure a functional stimulation of the graft thereby preventing its loss over time.

Current cleft treatment strategies are focused on infant orthopedics, post-operative pain relief, cleft maxillary hypoplasia, perioperative steroids, and alveoloplasty with no mention of dental rehabilitation [4]. With respect to dental rehabilitation of cleft patients, no standardized protocol has been put forward except by Takahashi [9]. The presented case is one of the first about immediately loaded dental implant in alveolar cleft site except for a retrospective analysis done by Garcia et al. [10]. Thus, prospective studies evaluating the success of immediately loaded dental implants in alveolar cleft sites are required so that patients’ treatment time can be reduced.

Cleft patients commonly develop mistrust and confusion due to a lack of guidance and knowledge about appropriate places for their treatment [11]. Presented case reports no such patient-related problems as all treatments starting from infancy till early adulthood were provided by a multidisciplinary team at a single center. This not only helped in gaining the patient’s trust in accepting the treatment and developed her confidence in the healthcare team but also ensured proper record-keeping. The healthcare team was not dependent on the patient for procuring medical history related to cleft treatment at any point during case management. The single center provided comprehensive care along with longitudinal and periodic assessments, something which is missing in cleft care rendered in India [12]. Considering the numerous esthetics concerns patients develop as they near adulthood, it is also important that a prosthodontist is part of the multidisciplinary team and is actively involved in treatment decisions.

## Conclusions

The case highlighted the importance of sequential treatment and the success of an immediately loaded dental implant in the cleft site. The authors propose undertaking trials involving dental implant-based rehabilitation of cleft patients and their immediate loading where possible. We also propose setting of multidisciplinary centers in regions where they are currently non-existent so that sequential treatment can be provided to patients along with meticulous record-keeping and longitudinal patient follow-up so that cleft care is not limited to urban localities but is also extended to interior geographical locations where the population is largely unaware.
